# Large-Scale Ichthyoplankton and Water Mass Distribution along the South Brazil Shelf

**DOI:** 10.1371/journal.pone.0091241

**Published:** 2014-03-10

**Authors:** Luis Carlos Pinto de Macedo-Soares, Carlos Alberto Eiras Garcia, Andrea Santarosa Freire, José Henrique Muelbert

**Affiliations:** 1 Laboratório de Crustáceos e Plâncton, Programa de Pós-graduação em Ecologia, Departamento de Ecologia e Zoologia, Universidade Federal de Santa Catarina, Florianópolis, Santa Catarina, Brazil; 2 Laboratório de Ecologia do Ictioplâncton, Instituto de Oceanografia, Universidade Federal do Rio Grande, Rio Grande, Rio Grande do Sul, Brazil; 3 Laboratório de Estudos dos Oceanos e Clima, Instituto de Oceanografia, Universidade Federal do Rio Grande, Rio Grande, Rio Grande do Sul, Brazil; The Evergreen State College, United States of America

## Abstract

Ichthyoplankton is an essential component of pelagic ecosystems, and environmental factors play an important role in determining its distribution. We have investigated simultaneous latitudinal and cross-shelf gradients in ichthyoplankton abundance to test the hypothesis that the large-scale distribution of fish larvae in the South Brazil Shelf is associated with water mass composition. Vertical plankton tows were collected between 21°27′ and 34°51′S at 107 stations, in austral late spring and early summer seasons. Samples were taken with a conical-cylindrical plankton net from the depth of chlorophyll maxima to the surface in deep stations, or from 10 m from the bottom to the surface in shallow waters. Salinity and temperature were obtained with a CTD/rosette system, which provided seawater for chlorophyll-a and nutrient concentrations. The influence of water mass on larval fish species was studied using Indicator Species Analysis, whereas environmental effects on the distribution of larval fish species were analyzed by Distance-based Redundancy Analysis. Larval fish species were associated with specific water masses: in the north, *Sardinella brasiliensis* was found in Shelf Water; whereas in the south, *Engraulis anchoita* inhabited the Plata Plume Water. At the slope, Tropical Water was characterized by the bristlemouth *Cyclothone acclinidens*. The concurrent analysis showed the importance of both cross-shelf and latitudinal gradients on the large-scale distribution of larval fish species. Our findings reveal that ichthyoplankton composition and large-scale spatial distribution are determined by water mass composition in both latitudinal and cross-shelf gradients.

## Introduction

Ichthyoplankton distribution is under the influence of environmental factors that regulate life history traits and determine its geographical range [1]–[3]. At large-scales, latitudinal gradients influence species diversity and composition in the marine realm (e.g., [4]–[6]) and most studies relate latitudinal influences directly with sea surface temperature (e.g., [4], [7]). In addition, latitudinal influences are also associated with oceanographic features such as water mass distribution (e.g., [8], [9]). Latitudinal influence is important in determining large-scale distribution, since fish larvae are dependent on temperature as well as long photoperiods for their development [7]. A latitudinal gradient is reported to be related to differences in larval assemblage composition in the North Pacific Ocean [10], to variability in egg size and newly hatched larval length of the anchovy *Engraulis rigens* in the Chilean coast [11], and to influence the growth rate and spawning season of the sea bass *Dicentrarchus labrax* in estuaries in Portugal [7].

It is known that larval fish assemblages result from adult spawning strategies and environmental influences [12], [3]. In the continental shelf and slope, ichthyoplankton species composition is strongly influenced by ocean currents and water masses [8], [13], [14]. Closer to the coast, continental runoff and coastal wind-induced upwelling play an important role in ichthyoplankton species composition [15], [13]. Therefore, the study of cross-shelf gradients in larval fish assemblages might help to understand the factors that influence larval species distribution and abundance.

The South Brazil Shelf (SBS), in the Southwest Atlantic Ocean (SWAO), is a region associated with the largest Brazilian fish stocks [15], [16], due to several coastal water enrichment processes. Continental runoff from La Plata estuary and Patos Lagoon [17]–[19] and both coastal and along shelf break upwelling [17], [20], [15] of the South Atlantic Central Water (SACW) are the main processes that influence nutrient availability in the euphotic zone [21]. In the Cape Frio and Cape Santa Marta region, coastal upwelling results in short duration and low intensity blooms, which increase phytoplankton and zooplankton biomass in this tropical region [17]. Submarine groundwater discharge might also provide an important flux of nutrients to the coastal waters due to the permeable sediments in the barriers of coastal lagoons along the southern Brazilian coast [22], [23]. These processes result in an increase in biological production that supports fisheries in this tropical/subtropical continental shelf.

Ichthyoplankton has been studied in the region over 30 years [24], focusing on larval recruitment of species of interest to fisheries (e.g., [25], [15]), the relationship between the distribution and composition of fish larvae and physical-chemical factors [8], [26], interactions between ichthyoplankton and zooplankton biovolume [27], and larval fish assemblages across a cross-shelf gradient [13], [28]. The patterns found are the result of adult distribution and reproductive strategies, water mass distribution, and zooplankton distribution, i.e., food availability [8], [13], [14]. Although cross-shelf gradients in larval fish assemblages have been studied in different oceans (e.g., [28], [14], [29], [30]), most experimental designs do not allow for concurrent cross-shelf and latitudinal gradient analysis. Therefore, the aim of this study was to investigate latitudinal and cross-shelf gradients in ichthyoplankton abundance to test the hypothesis that the large-scale distribution of fish larvae in the South Brazil Shelf is associated with water mass composition.

## Materials and Methods

### Study area

**Figure 1:**
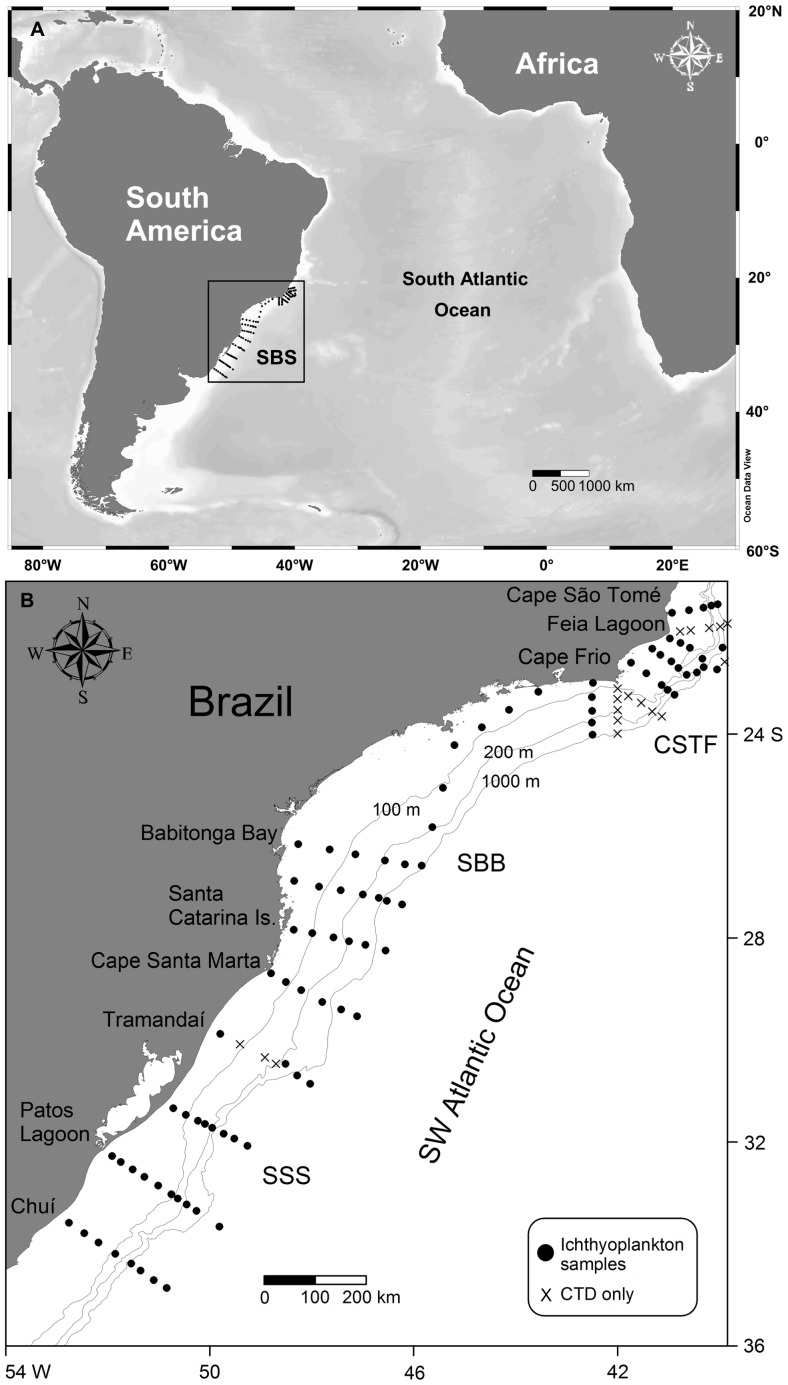
Map of the study region. A: Location of the study region on the South Atlantic Ocean (SBS, South Brazil Shelf inside the black square); B: Distribution of sampled stations in the South Brazil Shelf from Cape São Tomé (21°S) to Chuí (33°S) during the period of the cruise. CSTF, Cape São Tomé-Cape Frio region; SBB, Southern Brazilian Bight; SSS, Southern Subtropical Shelf.

The South Brazil Shelf (SBS), located at the Southwest Atlantic Ocean (SWAO) stretches from Cape São Tomé (21°S) to Chuí (33°S). The shelf waters of the SBS can be divided into three major latitudinal regions according to their oceanographic characteristics, from north to south: the Cape São Tomé-Cape Frio region (CSTF), the Southern Brazilian Bight (SBB) and the Southern Subtropical Shelf (SSS) (Figure 1A). Along the entire slope, warm and salty oligotrophic Tropical Water (TW) is transported southwards by the Brazil Current (BC) in the upper layer of the water column down to 200 m depth, whereas cold and nutrient-rich South Atlantic Central Water (SACW) occupies depths between 200 and 500 m [13], [31].

Coastal upwelling due to wind and topographic effects, occurs in the northern portion of the study area (CSTF) [32], [33], especially during austral spring and summer, due to prevalent northeasterly winds. The cold and nutrient-rich SACW is upwelled and provides special conditions for high primary production levels along the coastline. The region is also characterized by intense mesoscale activity [20] due to the proximity of the BC to the coast. The possibility of background eddy-induced upwelling is also suggested to exist, as the southward-flowing BC and its quasi-stationary unstable meanders approach the coast in the vicinity of Cape São Tomé [34]. The continental shelf is characterized by Shelf Water (SW) that results from the mixing of continental runoff with shelf waters influenced by the BC.

The Southern Brazilian Bight (SBB) is located between Cape Frio (22°52′S) and Cape Santa Marta (28°36′S), and is characterized by the presence of SW in its northern portion, and by Subtropical Shelf Water (STSW) in its southern area. The STSW is the result from a mixture of Plata Plume Waters (PPW) and TW, and spreads throughout the neritic region of the SBB and SSS [19]. In the SBB region, SACW contributes to high nitrate concentration mainly near Cape Frio and Cape Santa Marta [21], [35]. The SBB is also a biogeographic limit for the occurrence of reef fishes, since it is a transition area between tropical and temperate fauna [36]. Between Cape Santa Marta and the La Plata estuary (∼35°S), the SSS is characterized by the northwards spreading of the relatively cold and fresh Plata Plume Water (PPW). This transport is most intense during austral winter [19], when Subantarctic Shelf Water (SASW) is also transported northwards by the Patagonian Current (PC), which results in a wedge of cold water between PPW and STSW [19], [37]. A high silicate and phosphate concentration was associated with the terrestrial input from PPW and austral waters from SASW [21]. A sharp thermohaline frontal system exists between STSW and SASW around 33°S, named the Subtropical Shelf Front (STSF) [37], which increases local nutrient availability in the euphotic zone, primary production and zooplankton abundance [26].

### Data sampling and processing

Cruises were carried out between 21°27′S and 34°51′S during late austral spring (December 2010) and early austral summer (January 2011). Temperature, salinity, fluorescence and oxygen were measured vertically with a conductivity-temperature-depth (CTD) profiler Sea Bird Electronics model 911 at 107 stations distributed at 17 cross-shelf transects (Figure 1B). Additionally, water samples were collected with 5-L Niskin bottles to determine nutrients (ammonium, nitrite, nitrate, phosphate and silicate) and chlorophyll-a (chl-a) at selected depths (at 3 m or 5 m, the chlorophyll maximum depth and at the base of the mixture layer). Nutrients were determined using the methods described by Grasshoff et al. [38] and Strickland and Parsons [39], and chl-a according to Welschmeyer [40]. Vertical plankton tows were taken from the chlorophyll maximum depth to the surface at deep stations, and from 10 m from the bottom to the surface at homogeneous and shallow water stations (up to 20 m). Zooplankton samples were taken using a conical-cylindrical plankton net with a 200 µm mesh and 0.5 m mouth diameter, equipped with a digital flowmeter (General Oceanics) and towed behind the ship. The depth of the deep chlorophyll maximum ranged from 7 to 125 m and the depth of plankton samples ranged from 12 to 130 m. The mean (± SE) volume of water filtered by the net of all samples was 29.4±2.3 m^3^ and the values for each sample are displayed in Table S1. All necessary permits for the described field study were issued by Instituto Chico Mendes de Conservação da Biodiversidade (ICMBio/MMA), permit #13509-1 to ASF. All locations were not privately-owned or protected in any way, and the field study did not involve endangered or protected species.

Plankton samples (89, black circles in Figure 1B) were fixed and preserved in 4% buffered seawater-formaldehyde solution, since they are fragile and easily damaged and should be preserved immediately [41]. Ichthyoplankton was sorted and counted from the whole sample under a stereomicroscope. Fish eggs and larvae were identified to the lowest possible taxonomic level according to morphometric and meristic characteristics, using the larval development stages described by Ahlstrom and Moser [42], Bonecker and Castro [43], Fahay [44], Moser [45], Olivar and Beckley [46], Olivar and Fortuño [47], Olivar et al. [48] and Richards [49]. Additional literature was also used: Ciechomski [50], Derisio et al. [51], Matsuura [52], Matsuura and Suzuki [53], Moser et al. [54], Olivar and Beckley [55], Olney and Grant [56], and Sassa et al. [57]. Taxonomic classification was according to Nelson [58].

Sea surface temperature (SST) images were used to evaluate ichthyoplankton distribution in relation to surface thermal mesoscale features in the Brazilian shelf and slope waters, such as meanders, eddies and upwelling. We used monthly SST composition images for December 2010 and January 2011 with a 4 km spatial resolution from the Moderate Resolution Imaging Spectroradiometer (MODIS)/Aqua sensor. These images were obtained from the Ocean Color web site (http://oceancolor.gsfc.nasa.gov).

### Data analysis

Ichthyoplankton abundance was standardized to the number of individuals per 100 m^3^ filtered water. To select the most important species, a combination between the frequency of occurrence and relative abundance was obtained by multiplying these two values. The fish larvae species composition across the shelf was evaluated using samples classified according to a coast, inner shelf, outer shelf, inner slope or outer slope position. Between Cape São Tomé and Cape Frio, due to the narrow continental shelf, samples were classified as shelf. Chl-a and nutrients used in the analyses were integrated, whereas temperature and salinity were expressed as the mean value in the water column, from the depth of chl-a maxima to the surface (10 m depth). Oxygen stratification was used to characterize the relationship between ichthyoplankton abundance and oxygen distribution in the water column. This was calculated using the surface oxygen value and the value for the bottom of the oxycline, and respective depths.

Samples were also classified as being located within specific water masses (PPW, STSW, SW and TW) [19], [37] by their surface (10 m depth) temperature and salinity, since the majority of the samples were collected within these water mass depth ranges. South Atlantic Central Water was not used because its presence was not detected at the surface (10 m depth) in the region during the study. In addition, the physical control of ichthyoplankton species distribution was identified using Indicator Species Analysis (ISA) [59], according to groups formed by water mass classification. Multivariate Analysis of Variance with permutations (PERMANOVA) confirmed the validity of these groups formed *a priori* [60], using the Bray-Curtis index for similarity between samples. Species abundances were fourth-root transformed to reduce the weight of abundant species [61]. Prior to the analysis, the assumption of independence and homogeneity of multivariate dispersions within groups was tested using the PERMDISP routine. Once this assumption was confirmed (F = 2.527, p(perm) = 0.115), a one-way PERMANOVA design was used and whenever significant differences between groups were detected via PERMANOVA, they were tested using a pairwise test for comparisons among all pairs of groups [62]. PERMANOVA and additional tests were performed in PRIMER 6 with the PERMANOVA+ package [62], [63].

Distance-based Redundancy Analysis (db-RDA) was used to investigate the variability in ichthyoplankton composition constrained by centered environmental explanatory variables (latitude, distance from shore, temperature, salinity, oxygen stratification, ammonium, nitrite, nitrate, phosphate, silicate and chl-a), using the Bray-Curtis index for similarity between samples [64]. Prior to the analysis, ichthyoplankton abundance was Hellinger-transformed [65] to reduce the wide disparity in magnitude between species abundances. Only taxa that occurred in more than 4% of the samples were considered. The Cailliez correction was applied to the db-RDA function to avoid negative eigenvalues. The Variance Inflation Factor (VIF) was used to test and remove (VIF>20) collinearity between explanatory variables [66]. All explanatory variables were kept in the analysis according to the VIF. A Monte Carlo permutation test was used to test the null hypothesis of independence among species data and explanatory variables, and the significance of each canonical axis. Triplot was displayed using scaling = 2 and sample scores were plotted using the weighted sums of species scores [66]. Samples were also displayed according to specific water masses, using the same classification as Indicator Species Analysis. Distance-based Redundancy Analysis and additional tests were performed in R [67] with the Vegan and HH packages [68], [69].

## Results

### Oceanographic conditions

**Figure 2:**
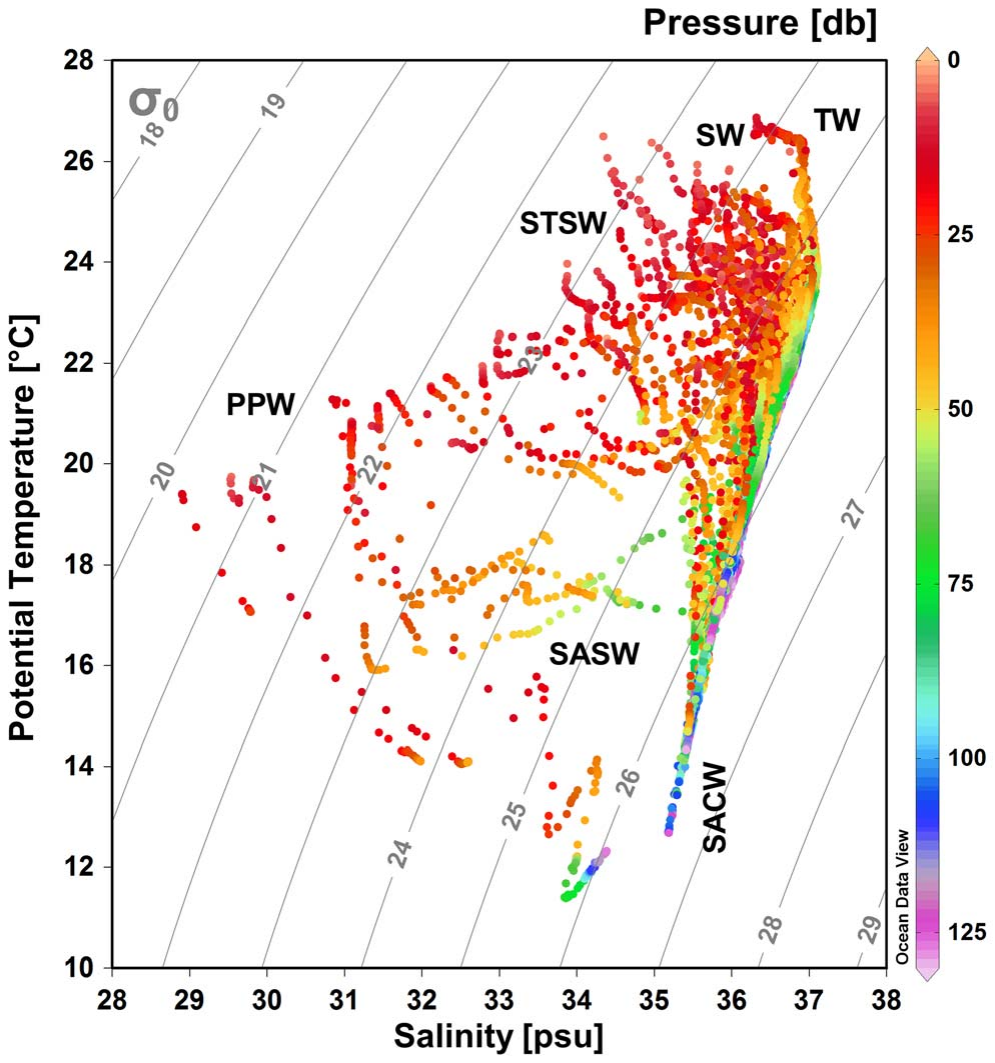
Temperature-salinity diagram from Cape São Tomé to Chuí for late austral spring and summer seasons. The water masses are as follows: Plata Plume Water (PPW), Subtropical Shelf Water (STSW), Shelf Water (SW), Tropical Water (TW), Subantarctic Shelf Water (SASW) and South Atlantic Central Water (SACW). Only stations to 130 m depth (maximum zooplankton sampling depth) are displayed.

Six water masses were identified in the study area down to the maximum zooplankton sampling depth (130 m): Subtropical Shelf Water (STSW), Shelf Water (SW), Tropical Water (TW), Plata Plume Water (PPW), Subantarctic Shelf Water (SASW) and South Atlantic Central Water (SACW) (Figure 2). Among these, three water masses were present in the shelf area (SW, STSW and PPW), except at stations near the coast in the Cape Frio-Cape São Tomé and Cape Santa Marta regions, where SACW was also found closer to the surface, and at stations located in the southern part of the study area (Chuí), where SASW was also present. At the slope, TW occupies the surface layer in the water column, whereas SACW is present at depths between 100 m and 500 m.

**Figure 3:**
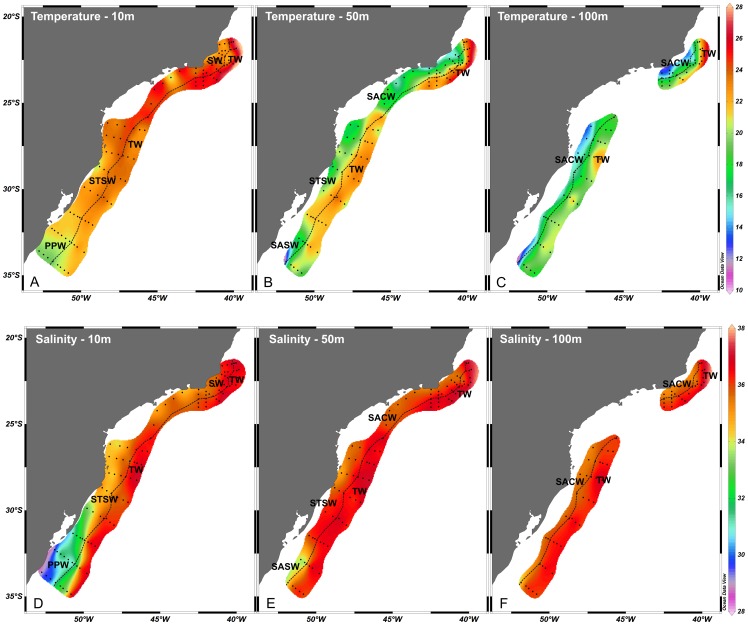
Temperarure and salinity horizontal distribution from Cape São Tomé to Chuí. A–C: Temperature at 10 m depth (A), 50 m depth (B) and 100 m depth (C); D–F: Salinity at 10 m depth (D), 50 m depth (E) and 100 m depth (F). The dashed black line represents approximately the shelf break position (∼200 m depth).

Over the shelf, a latitudinal pattern in the distribution of the water masses can be observed (Figure 3). Cold and fresh PPW spreads from Chuí to Tramandaí (∼30°S), mainly at 10 m depth. Due to its low density, PPW does not reach depths under 50 m, where it is replaced at its southern limit by SASW and at the northern limit by STSW. The area between Cape São Tomé and Cape Frio was occupied by warmer and salty SW, whereas STSW was the dominant water mass at the surface between Babitonga Bay and Tramandaí (25°S to 30°S). Salty-warm TW was present in the whole slope area from the surface to a depth of 100 m. Intrusion of SACW was evident from Cape São Tomé (21°S) to Cape Santa Marta (28°S) at 50 m and 100 m depth, reaching lower depths (∼20–30 m) in the vicinity of Cape Frio and Cape Santa Marta.

### Ichthyoplankton composition and distribution

A total of 1,447 fish larvae and 1,930 fish eggs were caught during the cruise. Larval identification resulted in 115 taxa that belonged to 40 families (Table S2), and 52 of these taxa were caught in only one sample. Ninety-six taxa were representative of an individual species. Identification occurred at the species level for 58 of these 96 taxa, and 38 were identified to the family or genus level (e.g., Bythitidae sp. and *Auxis* sp.). The 19 remaining taxa might be members of two or more species (e.g., Macrouridae spp.). The most specious family in the samples was Myctophidae (21), followed by Serranidae (5), Paralepididae (4), Scorpaenidae (4), Carangidae (4), Sciaenidae (4) and Scombridae (4) (Table S2). Fish egg identification resulted in three taxa. Engraulidae eggs were the most abundant and represented 21% of the total eggs caught. However, 75% off eggs remained unidentified (Table S2).

**Figure 4:**
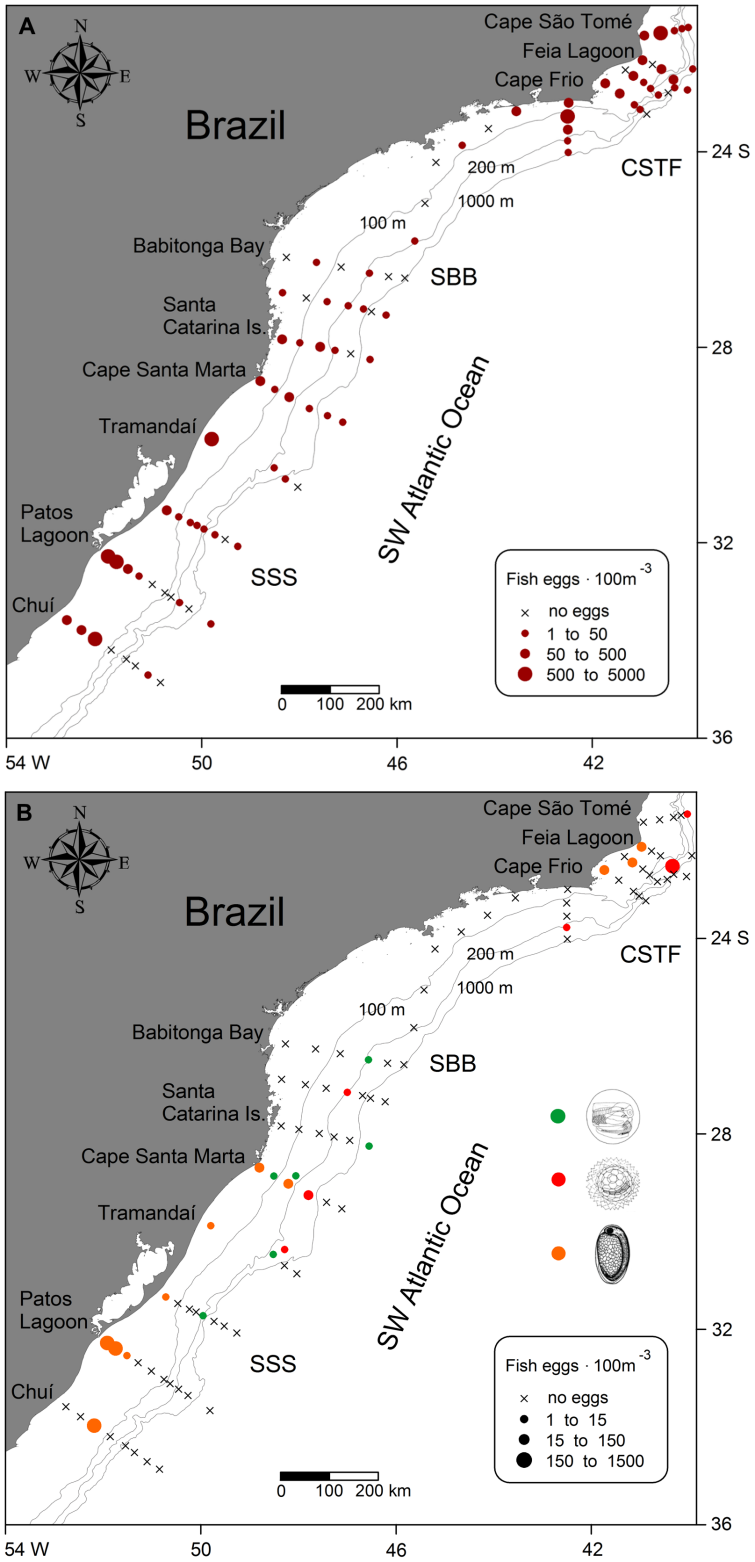
Fish egg abundance distribution at the South Brazil Shelf. A: Distribution of total fish egg abundance; B: Distribution of Anguilliformes eggs (green circles), *Maurolicus muelleri* eggs (red circles) and Engraulidae eggs (orange circles) abundances. CSTF, Cape São Tomé-Cape Frio region; SBB, Southern Brazilian Bight; SSS, Southern Subtropical Shelf.

Fish egg distribution showed spawning activity in the whole area, mostly at coastal and shelf stations (Figure 4A). Peaks in fish egg abundance were found in front of Patos Lagoon (4,116 ind. 100 m^−3^), on the coast of Tramandaí (631 ind. 100 m^−3^) and on Cape Frio (1,174 ind. 100 m^−3^) and Cape São Tomé (1,184 ind. 100 m^−3^) shelves. The abundance of Engraulidae eggs was higher near Patos Lagoon (1,403 ind. 100 m^−3^) and Anguilliformes eggs showed a low abundance and were mostly present in the SBB area. The abundance of *Maurolicus muelleri* eggs peaked at the slope near to the Feia lagoon area (374 ind. 100 m^−3^) and occurred along the slope in the southerly direction (Figure 4B).

**Figure 5:**
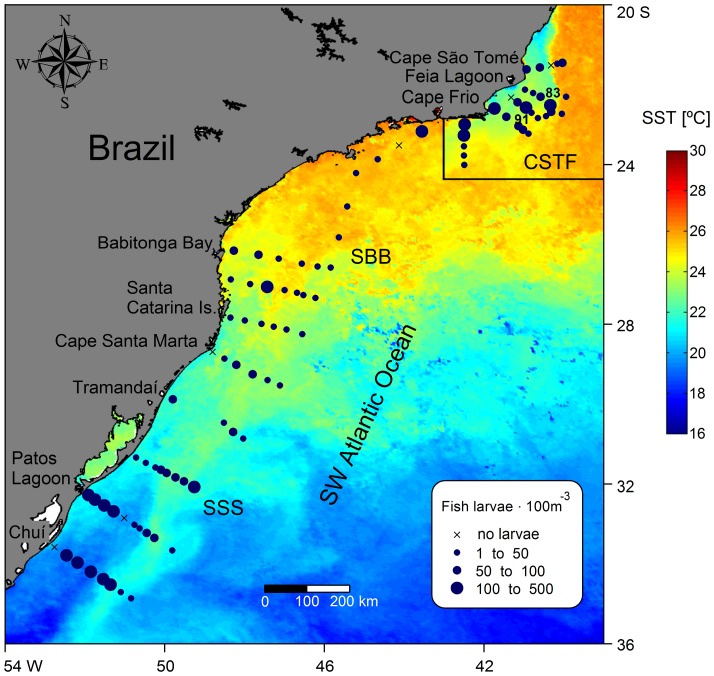
Fish larvae abundance distribution at the South Brazil Shelf. Fish larvae abundance (blue circles) superimposed onto a SST satellite image monthly composition, from December 2010. Inside the square, the monthly SST image from January 2011 is shown. CSTF, Cape São Tomé-Cape Frio region; SBB, Southern Brazilian Bight; SSS, Southern Subtropical Shelf.

Fish larvae abundance was higher at SSS (Figure 5), mainly near Chuí (168 to 445 ind. 100 m^−3^) and the Patos Lagoon area (119 to 479 ind. 100 m^−3^), the slope near Feia Lagoon (215 ind. 100 m^−3^) and the shelf at Cape Frio (131 to 246 ind. 100 m^−3^). Low abundances were observed mostly at the slope, nevertheless, two peaks of high abundance were registered in the northern area at stations 83 and 91 (Figure 5). In station 91, situated on the outer shelf (103 ind. 100 m^−3^), there was a mixture of yolk sac larvae (29%), larvae of Scorpaenidae sp. 1 (14%) and larvae of pelagic fishes such as Myctophidae (29%), Phosichthyidae (14%) and Paralepididae (7%). Station 83, located at the slope (215 ind. 100 m^−3^), the same stations where *M. muelleri* eggs peaked (Figure 4B), mainly contained yolk sac larvae (204 ind. 100 m^−3^). The SST image showed the formation of BC meanders at the boundary between TW at the slope and SW over the continental shelf in the northern area (Figure 5), and this is coincident with the position of stations 83 and 91 position in the area.

Engraulidae larvae were the most abundant (35%) followed by those of Myctophidae (24%), Clupeidae (9%), Bregmacerotidae (3%), Scombridae (3%) and Gobiidae (3%). The highest abundance was for the anchovy *Engraulis anchoita* at 17.3±7.1 ind. 100 m^−3^ and occurred in 18% of all samples. The second most abundant was the lanternfish *Diaphus brachycephalus* (6.3±2.5 ind. 100 m^−3^) with a 22% frequency of occurrence, followed by the Brazilian sardine *Sardinella brasiliensis* (4.7±2.9 ind. 100 m^−3^). Some species had a low abundance but were frequently found in samples, for example, larvae of the pelagic fishes such as the lanternfishes *Diaphus mollis* (26%) and *Ceratoscopelus townsendi* (13%); the bristlemouth *Cyclothone acclinidens* (16%); the codlet *Bregmaceros cantori* (10%); and others from coastal origins such as the lizardfish *Synodus foetens* (10%) and the largehead hairtail *Trichiurus lepturus* (10%) (Table S2).

**Figure 6:**
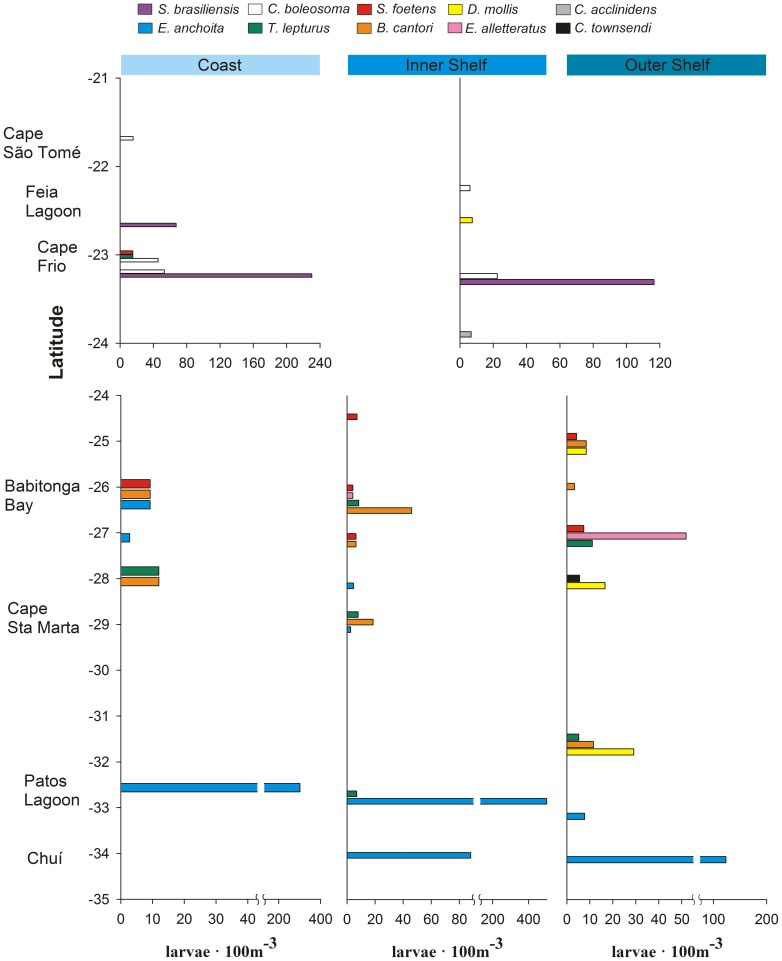
Latitudinal and cross-shelf distribution of ichthyoplankton composition at the shelf of the South Brazil Shelf. Fish larvae composition of the 10 most important species according to the combination of relative abundance and frequency are shown for the coast and the inner and outer shelf. Stations between 21°S and 24°S latitude were classified only as shelf because of the narrow continental shelf. Note the difference of scales on the X axes. Full species names are given in Table S2.

**Figure 7:**
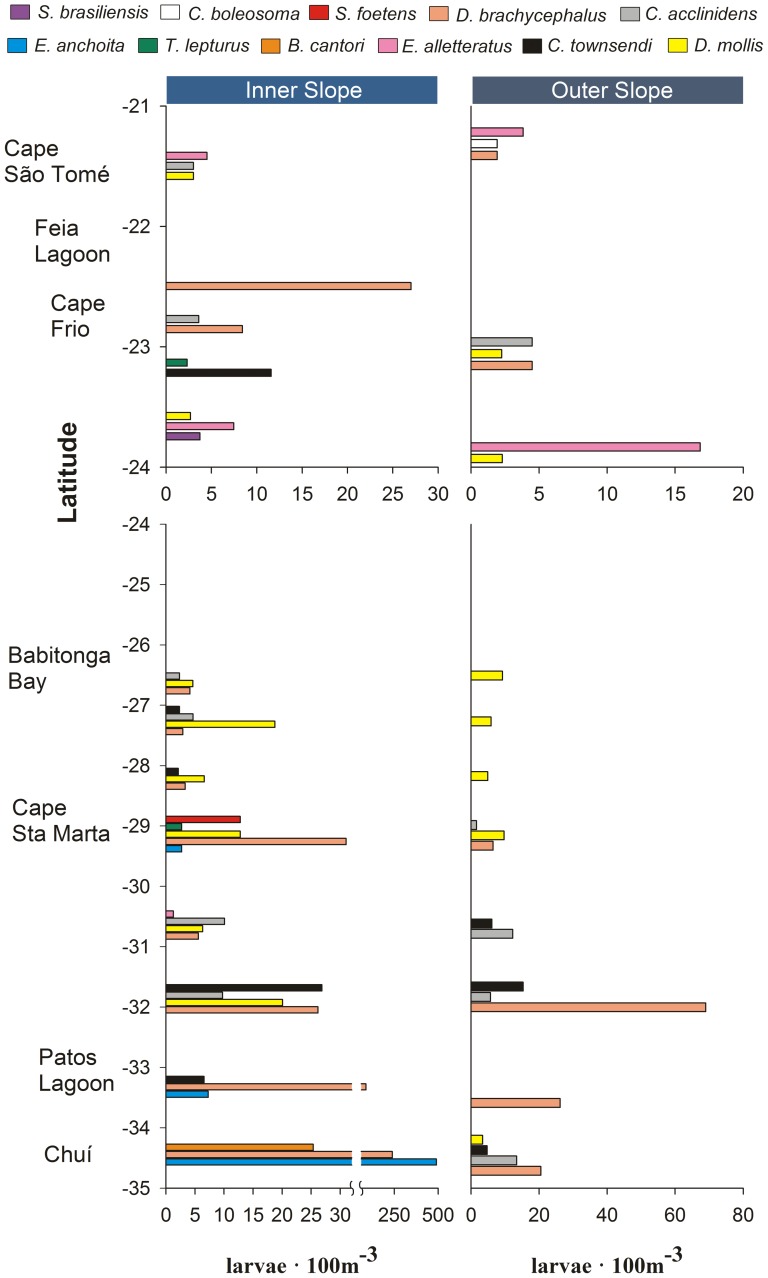
Latitudinal and cross-shelf distribution of ichthyoplankton composition at the slope of the South Brazil Shelf. Fish larvae composition of the 11 most important species according to the combination of relative abundance and frequency are shown for the inner and outer slope. Note the difference of scales on the X axes. Full species names are given in Table S2.

A turnover was observed in both dominant species according to the latitude in the shelf and in species composition according to a cross-shelf direction (Figure 6, 7). At high latitudes and over the shelf *E. anchoita* was the dominant species. Between 25°S and 30°S, anchovy shared dominance with *T. lepturus*, *S. foetens* and *B. cantori* in the coast and inner shelf. *Trichiurus lepturus* and *S. foetens* also occurred between Cape São Tomé and Cape Frio, but *S. brasiliensis* and the goby *Ctenogobius boleosoma* were the most abundant species on the shelf (Figure 6). Larvae of pelagic species such as the tuna *Euthynnus alletteratus* and the lanternfishes *D. mollis* and *C. townsendi* occurred on the outer shelf and inner slope area at all latitudes (Figure 6, 7). At the slope, *E. alletteratus* remained abundant between Cape São Tomé and Cape Frio, and the lanternfishes including *D. brachycephalus*, together along with the bristlemouth *C. acclinidens*, dominated the slope area between 26°S and 35°S (Figure 7).

### Fish larvae assemblages and water masses

According to Indicator Species Analysis (ISA), the group formed by SW samples was characterized by larvae of the Brazilian sardine *S. brasilliensis*, the goby *C. boleosoma*, and the blenny *Parablennius* sp., which resulted in an assemblage with a mix of reef-associated/benthic species (blennies and gobies) and pelagic species (sardines), considering adult habitat (Table 1). In the STSW group, the larvae of two fishes of coastal provenance; the largehead hairtail *T. lepturus* and the lizardfish *S. foetens*, and larvae of the codlet *B. cantori* were the indicator species. Furthermore, larvae of the anchovy *E. achoita* and Engraulidae eggs characterized the group from the PPW. The TW group was characterized by the bristlemouth *C. acclinidens*. All groups formed by the four water masses are valid according to PERMANOVA (Pseudo-F = 4.095, p(perm) = 0.0001, Residual d.f. = 77), when all mean similarities within groups were higher than similarities between groups (Table 2).

**Table 1:** Results of indicator species analysis (ISA), indicating groups according to the water mass in which the sample was collected in the South Brazil Shelf.

Indicator species	Indicator	Monte Carlo p value^1^	Water mass^2^
	value (%)		
*Sardinella brasiliensis*	23.5	0.007	SW
*Ctenogobius boleosoma*	29.2	0.004	SW
*Parablennius* sp.	11.8	0.040	SW
*Bregmaceros cantori*	35.7	0.002	STSW
*Trichiurus lepturus*	32.0	0.002	STSW
*Synodus foetens*	30.5	0.002	STSW
*Engraulis anchoita*	56.5	0.001	PPW
Engraulidae eggs	27.9	0.017	PPW
*Cyclothone acclinidens*	27.6	0.011	TW

1 Only species with significant results (p<0.05) according to the Monte Carlo test are shown.

2 Water masses: SW, Shelf Water; STSW, Subtropical Shelf Water; PPW, Plata Plume Water; TW, Tropical Water.

**Table 2:** Results of PERMANOVA pairwise tests indicating differences between groups formed by water mass classification in the South Brazil Shelf.

PERMANOVA pairwise tests		
Groups^1^	Similarity^2^	P(perm)
SW-STSW	4.813	0.0009
SW-PPW	3.645	0.0001
SW-TW	4.012	0.0001
STSW-PPW	10.987	0.0116
STSW-TW	4.399	0.0001
PPW-TW	4.744	0.0001

1 Water masses: SW, Shelf Water; STSW, Subtropical Shelf Water; PPW, Plata Plume Water; TW, Tropical Water.

2 Similarity between groups. Similarity within groups: SW = 4.935, STSW = 15.978, PPW = 15.472, TW = 14.990.

### Environmental effects on the distribution of larval fish assemblages

**Figure 8:**
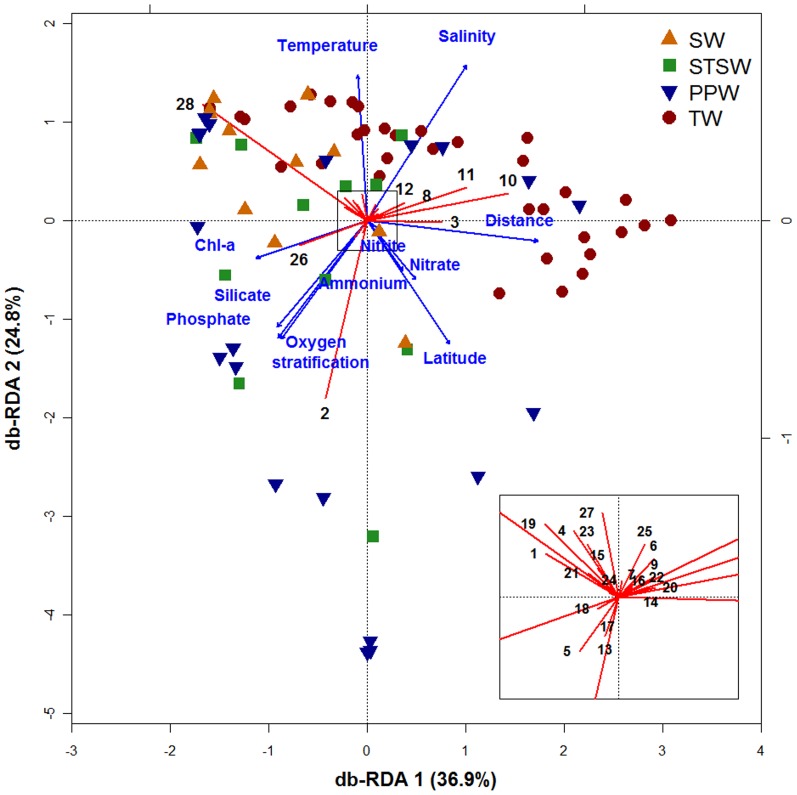
Distance-based Redundancy Analysis ordination for ichthyoplankton composition constrained by environmental variables. Triplot with explanatory variables, species and samples. Numbers represents species/taxa names: 1. *Sardinella brasiliensis*; 2. *Engraulis anchoita*; 3. *Cyclothone acclinidens*; 4. *Pollichthys mauli*; 5. *Synodus foetens*; 6. *Lestrolepsis intermedia*; 7. *Myctophum nitidulum*; 8. *Ceratoscopelus townsendi*; 9. *Diaphus garmani*; 10. *Diaphus brachycephalus*; 11. *Diaphus mollis*; 12. *Lepidophanes guentheri*; 13. *Bregmaceros cantori*; 14. *Urophycis mystacea*; 15. Scorpaenidae sp. 1; 16. *Prionotus* sp.; 17. *Bairdiella* sp.; 18. *Micropogonias furnieri*; 19. *Ctenogobius boleosoma*; 20. *Sphyraena barracuda*; 21. *Trichiurus lepturus*; 22. *Auxis* sp.; 23. *Euthynnus alletteratus*; 24. *Scomber colias*; 25. Anguilliformes eggs; 26. Engraulidae eggs; 27. *Maurolicus muelleri* eggs; 28. Other fish eggs.

Distance-based Redundancy analysis (db-RDA) constrained 24% of ichthyoplankton variance in relation to the explanatory variables (Figure 8). The Monte Carlo permutation test was significant for all canonical axes together (F = 1.926, p = 0.001) and rejected the null hypothesis of independency between ichthyoplankton and environmental variables. In addition, the first and second canonical axes were significant and accounted together for 61.7% of the constrained variance. The first axis (db-RDA 1, F = 7.808, p = 0.001), accounted for 36.9% of the variance, represented the cross-shelf gradient, and was positively correlated with distance from shore (0.784) and salinity (0.459); and was negatively correlated with chl-a (−0.511). However, the second axis (db-RDA 2, F = 5.264, p = 0.001), which accounted for 24.8%, represented the latitudinal gradient (Figure 8), was positively correlated with salinity (0.716) and temperature (0.669); and was negatively correlated with latitude (−0.569), oxygen stratification (−0.545), phosphate (−0.539) and silicate (−0.488).

According to the species distribution (Figure 8), larvae of the anchovy *E. anchoita* were positively correlated with latitude, oxygen stratification, silicate and phosphate concentration and low temperatures from PPW. At mid-latitudes and in inshore waters, under the influence of STSW, larvae of the codlet *B. cantori*, the largehead hairtail *T. lepturus* and the lizardfish *S. foetens* were also positively correlated with high chl-a concentration. The abundance of Engraulidae eggs and other fish eggs was highest mostly in inshore waters, with a high chl-a concentration. In addition, a high abundance of *S. brasiliensis* and *C. boleosoma* larvae and *M. muelleri* eggs was influenced by the high-temperature SW. Larvae of oceanic species, such as the bristlemouth *C. acclinidens* and the lanternfishes *D. mollis*, *D. brachycephalus*, *L. guentheri and C. townsendi* were associated with TW, low nutrients and chl-a concentration, and high salinity.

## Discussion

Our findings provide information on ichthyoplankton distribution and its relationship with the oceanographic environment within a wide latitudinal range in the South Brazil Shelf. Four surface water masses were present in the whole study area, and over the shelf, three were positioned in a latitudinal gradient. Larval fish species were associated with specific water masses: in the north *Sardinella brasiliensis* characterized Shelf Water (SW); in the south *Engraulis anchoita* was found in Plata Plume Water (PPW); and, in Subtropical Shelf Water (STSW) species from both coastal and shelf origins were found. At the slope, Tropical Water (TW) was characterized only by the bristlemouth *Cyclothone acclinidens*, although a high abundance and occurrence of lanternfishes were also found. Ichthyoplankton composition was influenced by the cross-shelf gradient and by the latitude in continental shelf waters.

A maximum ichthyoplankton abundance and the presence of coastal- and estuarine-related species, such as *Parona singnata*, Gerreidae, *Cynoscion* sp., *Micropogonias furnieri*, *Mugil* sp., *Symphurus* spp. and Engraulidae eggs, are expected near or in front of large estuaries because of the contribution of estuarine spawners and estuarine-dependent fishes that use these environments for reproduction and/or as nursery areas [70]–[72]. Even small coastal lagoons can contribute to high quantities of fish eggs and larvae and increase ichthyoplankton abundance in coastal waters [73]. Continental runoff and tidal cycles associated with wind stress transport fish eggs and larvae to coastal waters [74] and increase their abundance in adjacent regions. Another process that might increase ichthyoplankton abundance in the continental shelf is the wind induced coastal upwelling (e.g., [15], [3]). In Brazilian shelf waters, areas under the influence of coastal upwelling, such as Cape São Tomé and Cape Frio [35]), showed a high abundance of eggs (>1000 ind. 100 m^−3^) and larvae (>100 ind. 100 m^−3^), once the enhancement of the primary and secondary production ensure the availability of food for the future larvae [27], [33]. This is especially required during the critical period [75].

Ichthyoplankton abundance distribution in shelf waters was comparable to that of previous studies in the region between Cape Frio and Chui [8], [27]. In addition, a high abundance of fish larvae in the southernmost of the study area is due to freshwater influences by PPW [26], and is comparable to other temperate and oligotrophic areas in the south-western Australian coast [14], where a maximum larval abundance of 414 ind. 100 m^−3^ in late austral spring was found. Fish egg abundance was similar, and sometimes higher, than that described for the Northern Brazilian coast [76]. The abundance of eggs and larvae in oceanic waters was lower (<50 ind. 100 m^−3^) than in coastal and shelf waters, and fish eggs were absent in some offshore stations. This is probably an effect of the nutrient-poor TW that is driven by the Brazil Current (BC) throughout the entire slope [19], [31]. In most of the slope stations, the abundance of fish larvae followed the trend described for oceanic regions [77], [78].

A high larval abundance occurred between Cape São Tomé and Cape Frio, at the outer shelf and slope. This pattern might be related to the eddy-induced upwelling [34] that contributes to the enrichment of the euphotic layer by intrusion of the South Atlantic Central Water (SACW) and consequently to shelf brake upwelling [20]. In addition, peaks in fish larvae abundance at the slope are possibly due to larval retention by meanders or eddies from BC [35], [8]. This mechanism of larval retention was described in the Mediterranean Sea, when a high concentration of tuna larvae inside anti-cyclonic eddies induced by topography was indicative that these structures act as retention areas for these larvae [3].

Fish larvae species composition reflected the spawning area of adult fish populations and was similar to that previously registered for the area [8], [24]. Coastal and shelf waters showed a dominance of larvae from coastal species such as Trichiuridae and/or of larvae from demersal eggs such as those of Gobiidae, Blenniidae, and Synodontidae. This region also contained species from the continental shelf and slope with pelagic eggs such as those of Clupeidae, Engraulidae, Bregmacerotidae and Scombridae. Some oceanic species spawn near land masses, such as tuna larvae *Auxis* sp., *Thunnus* spp. and *Katsuwonus pelamis* and release their eggs in areas with a higher primary production than in the open ocean, where waters are more suitable for the early larvae [79]. However, our results showed that these species were distributed in different parts of the shelf in a latitudinal gradient according to their specific water mass. Alternatively, larvae of oceanic fish such as the Myctophidae and Gonostomatidae occupied mostly the inner and outer slope in the entire study area, as previously shown [8], [24]. Since most of these species have a circumglobal distribution, this pattern has been recorded in different oceanic areas around the world (e.g., [77], [80], [78], [14], [81]).

Water mass composition is an important controlling factor that conditions larval fish distribution [80], [14], [81]. The water mass composition in the southern part (SSS) of the study area was very similar to that demonstrated previously for the area [19], [37] and influenced fish larvae species distribution. The SW group was influenced by a mixture of larvae from demersal eggs, such as those of the goby *C. boleosoma* and the blenny *Parablennius* sp., with larvae from pelagic eggs such as from the sardine *S. brasiliensis*. In a region in southwestern Australia, inshore stations were also dominated by larvae from demersal eggs such as those from Tripterygiidae, Gobiidae and Blenniidae, and larvae of neritic species such as Clupeidae [14].

The spawning area of the Brazilian sardine *S. brasiliensis* is located on the Southern Brazilian Bight (SBB) between Cape Frio (∼23°S) and Cape Santa Marta (∼28°S) [25], [82]. Sardine spawning is strongly dependent on and influenced by SACW intrusion and the stability of the water column due to vertical stratification [83], which is a key ingredient for larval survival [84], since it helps larval retention and nutrient enrichment of the euphotic layer [83], [25]. In this study, the intrusion of the SACW over the inner shelf occurred in the vicinity of Cape Santa Marta and Cape Frio due to local coastal wind-induced upwelling [85], [32]. A close inspection of the isotherms in cross-shelf sections showed a more pronounce intrusion of SACW in Cape Frio (∼20–30 m depth), which increased the stratification of the water column, favoring the occurrence of *S. brasiliensis* in the continental shelf area around Cape Frio.

The STSW group comprised mainly stations from the SBB area, and was associated with larvae of fish that spawn in inshore waters, such as the lizardfish *S. foetens* and the largehead hairtail *T. lepturus*, which shared dominance with larvae of oceanic origin such as the codlet *B. cantori*. Due to the lack of intrusion of SACW in the SBB area, the STSW group did not contain any *S. brasiliensis* larvae in its composition. This same larval association was registered in the Subtropical Shelf Front (STSF), the transition between the STSW and Subantartic Shelf Water (SASW) located near the 50 m isobaths, around 33°S [26], [37]. This frontal assemblage was studied in the summer and was characterized by the occurrence of species of both coastal or oceanic origin, such as *T. lepturus* and other codlet *B. atlanticus*, respectively [26].

In the area under the influence of PPW, larvae of the anchovy *E. anchoita* and its pelagic eggs were dominant. Anchovy has a wide distribution range from Cape Frio (23°S) to the Gulf of San Jorge (47°S) [86], and unlike other anchovy species that form large schools in areas under the influence of intense upwelling [82], the distribution of *E. anchoita* is associated with a lower intensity upwelling, cold water from the south and continental runoff from La Plata and Patos Lagoon [87], [88], [19]. It is the only species that occurred in both winter and summer assemblages, and in inshore, frontal and offshore assemblages at the STSF [26]. Its wide range distribution and capacity to survive in different oceanographic conditions make *E. anchoita* larvae an important component of the pelagic ecosystem in the Southwest Atlantic Ocean (SWAO).

Regarding the TW group, characterized by a high abundance and occurrence of the bristlemouth *C. acclinidens*, Myctophidae species such as *D. mollis*, *D. brachycephalus* and *C. townsendi* were also overwhelmingly abundant in the slope waters. Larvae from Myctophidae and Gonostomatidae are good indicators of the presence of oceanic waters such as TW [80], which partly explained the occurrence of oceanic species over the shelf, which can result from the intrusion of TW through the continental shelf. Water masses were good predictors of larval species associations that resulted in significantly different assemblages from each other, as in this study. In fact, species associations from water masses can be stronger than those that are only considered a spatial or a temporal association [14].

The main role of water mass influence can be attributed to the ordination of larval species distribution in space. Cross-shelf and latitudinal environmental effects were the two main components that accounted for the composition of fish larval assemblages, and reflect the influence of water masses (e.g., [8], [14], [9]). The latitudinal gradient shows an inverse relationship with sea surface temperature (e.g., [4], [7]), whereas cross-shelf variability is related to salty ocean waters and coastal productive waters (e.g., [13]). In the oligotrophic Brazil Current domain, upwelling of SACW is the main input of nitrates over the continental shelf, whereas phosphates and silicates might derive from terrestrial origin and be associated with the PPW contribution [21]. This is the main characteristic observed in our cruises (Figure 8), where phosphorous and silicate are present at high latitudes and indicate the influence of continental runoff.

Our results revealed a high unexplained source of variation, and we suggest that other sources of variability should be investigated. Large-scale ichthyoplankton composition and distribution are also influenced by food availability and geostrophic circulation [75], [84], [2], and we were not able to assess these. Coupling between physical–chemical and biological effects are an important mechanism in larval fish growth and survival. For example, SBB restricted the *S. brasiliensis* population and its spawning is influenced by SACW intrusion [15]. Studies from Matsuura [15] showed that the failures in recruitment of two year-classes (1975 and 1987) was probably because the lack of intrusion of SACW in the SBB inner shelf, which caused a high mortality of sardine larvae [15], [82]. In the Mediterranean Sea, shelf species are mainly influenced by trophic resources, whereas oceanic species are controlled by current-mediated transport [2].

In conclusion, our study supports the hypothesis that in the South Brazil Shelf, the large-scale distribution of the ichthyoplankton is mainly controlled by water mass composition. In addition, larval fish assemblages are influenced by the cross-shelf gradient and by the latitude of continental shelf waters. Species are more abundant and frequent at different areas under the influence of specific thermohaline characteristics in the water column. Further studies should be performed to assess the role of food availability in the control of fish larvae abundance.

## Supporting Information

Table S1 **Plankton data for the stations visited at the South Brazil Shelf.** Depth of plankton sample (m) and water volume (m^3^) filtered for each of the 89 stations between Cape São Tomé (21°S) and Chuí (33°S) from December 2010 to January 2011.
(DOC)

Table S2 **Taxonomic list of the ichthyoplankton from the South Brazil Shelf.** Total catch of fish larvae and eggs, abundance (mean ± SE) and frequency (%) for the 89 stations visited between Cape São Tomé (21°S) and Chuí (33°S) from December 2010 to January 2011.
(DOC)
