# Supplementary material for: Large-Scale Ichthyoplankton and Water Mass Distribution along the South Brazil Shelf
Source: PLoS One. 2014 Mar 10;9(3):e91241. doi: 10.1371/journal.pone.0091241 (PMC3948790; doi:10.1371/journal.pone.0091241)
Supplement: Table S1 — Plankton data for the stations visited at the South Brazil Shelf. Depth of plankton sample (m) and water volume (m3) filtered for each of the 89 stations between Cape São Tomé (21°S) and Chuí (33°S) from December 2010 to January 2011. (DOC) [file pone.0091241.s001.doc]

**Table S1. Plankton data for the stations visited at the South Brazil Shelf.** Depth of plankton sample (m) and water volume (m^3^) filtered for each of the 89 stations between Cape São Tomé (21ºS) and Chuí (33ºS) from December 2010 to January 2011.

| Station | Plankton sample depth (m) | Water volume (m^3^) |
| --- | --- | --- |
| 1 | 23 | 5.52 |
| 2 | 22 | 2.27 |
| 3 | 25 | 11.33 |
| 4 | 30 | 7.87 |
| 5 | 40 | 8.39 |
| 6 | 30 | 3.94 |
| 7 | 85 | 63.25 |
| 8 | 87 | 29.30 |
| 9 | 120 | 80.17 |
| 10 | 115 | 50.90 |
| 11 | 95 | 38.21 |
| 12 | 25 | 13.72 |
| 13 | 30 | 38.80 |
| 14 | 31 | 17.82 |
| 15 | 32 | 14.29 |
| 16 | 30 | 29.65 |
| 17 | 17 | 14.53 |
| 18 | 13 | 7.43 |
| 19 | 39 | 20.76 |
| 20 | 40 | 14.74 |
| 21 | 80 | 77.34 |
| 22 | 70 | 35.58 |
| 23 | 100 | 23.91 |
| 24 | 100 | 33.04 |
| 25 | 105 | 29.82 |
| 26 | 110 | 52.15 |
| 27 | 120 | 65.26 |
| 28 | 105 | 79.07 |
| 29 | 40 | 35.84 |
| 34 | 25 | 9.50 |
| 35 | 14 | 5.09 |
| 36 | 58 | 37.60 |
| 37 | 68 | 37.19 |
| 38 | 70 | 42.07 |
| 39 | 85 | 61.95 |
| 40 | 100 | 34.80 |
| 45 | 22 | 8.36 |
| 46 | 50 | 21.36 |
| 47 | 38 | 18.02 |
| 48 | 45 | 46.70 |
| 49 | 82 | 30.21 |
| 50 | 107 | 40.38 |
| 51 | 89 | 51.16 |
| 52 | 92 | 42.65 |
| 53 | 75 | 42.60 |
| 54 | 61 | 34.61 |
| 55 | 30 | 26.99 |
| 56 | 48 | 15.49 |
| 57 | 31 | 36.21 |
| 58 | 25 | 10.83 |
| 59 | 37 | 23.90 |
| 60 | 49 | 19.72 |
| 61 | 66 | 24.24 |
| 62 | 85 | 43.11 |
| 63 | 85 | 21.59 |
| 64 | 86 | 30.11 |
| 65 | 39 | 23.58 |
| 66 | 36 | 13.98 |
| 67 | 37 | 14.97 |
| 68 | 30 | 10.14 |
| 69 | 32 | 11.30 |
| 70 | 12 | 6.37 |
| 71 | 22 | 7.35 |
| 72 | 13 | 7.10 |
| 73 | 60 | 66.29 |
| 74 | 66 | 52.11 |
| 80 | 14 | 4.27 |
| 81 | 32 | 16.58 |
| 82 | 29 | 11.11 |
| 83 | 21 | 8.83 |
| 84 | 121 | 61.46 |
| 86 | 130 | 48.68 |
| 87 | 25 | 8.98 |
| 88 | 50 | 28.79 |
| 89 | 75 | 55.30 |
| 90 | 39 | 23.76 |
| 91 | 46 | 13.54 |
| 92 | 22 | 11.17 |
| 93 | 22 | 10.76 |
| 94 | 20 | 25.23 |
| 95 | 35 | 13.82 |
| 96 | 55 | 24.58 |
| 97 | 90 | 43.10 |
| 98 | 89 | 44.44 |
| 108 | 78 | 130.58 |
| 109 | 98 | 37.07 |
| 110 | 60 | 26.81 |
| 111 | 40 | 22.31 |
| 112 | 22 | 6.56 |
