# Supplementary material for: Large-Scale Ichthyoplankton and Water Mass Distribution along the South Brazil Shelf
Source: PLoS One. 2014 Mar 10;9(3):e91241. doi: 10.1371/journal.pone.0091241 (PMC3948790; doi:10.1371/journal.pone.0091241)
Supplement: Table S2 — Taxonomic list of the ichthyoplankton from the South Brazil Shelf. Total catch of fish larvae and eggs, abundance (mean ± SE) and frequency (%) for the 89 stations visited between Cape São Tomé (21°S) and Chuí (33°S) from December 2010 to January 2011. (DOC) [file pone.0091241.s002.doc]

**Table S2.** **Taxonomic list of the ichthyoplankton from the South Brazil Shelf.** Total catch of fish larvae and eggs, abundance (mean ± SE) and frequency (%) for the 89 stations visited between Cape São Tomé (21ºS) and Chuí (33ºS) from December 2010 to January 2011.

| Family | Species | Total catch (nº. of larvae) | Abundance ± SE (nº. ∙ 100m^-3^) | Frequency (%) |
| --- | --- | --- | --- | --- |
| Congridae | *Ariosoma balearicum* (Delaroche, 1809) | 2 | 0.16±0.14 | 2.25 |
| Ophichthidae | *Ophichthus gomesii* (Castelnau, 1855) | 1 | 0.03±0.03 | 1.12* |
| Clupeidae | *Sardinella brasiliensis* (Steindachner, 1879) | 70 | 4.69±2.97 | 4.49 |
| Engraulidae | *Engraulis anchoita* Hubbs and Marini, 1935 | 240 | 17.33±7.15 | 17.98 |
| Gonostomatidae | *Cyclothone acclinidens* Garman, 1899 | 38 | 0.87±0.25 | 15.73 |
| Gonostomatidae | *Cyclothone alba* Brauer, 1906 | 7 | 0.12±0.08 | 3.37 |
| Gonostomatidae | *Cyclothone pseudopallida* Mukhacheva, 1964 | 3 | 0.09±0.08 | 2.25 |
| Gonostomatidae | *Cyclothone* spp. | 14 | 0.26±0.1 | 8.99 |
| Sternoptychidae | *Maurolicus muelleri* (Gmelin, 1789) | 1 | 0.15±0.15 | 1.12* |
| Phosichthyidae | *Pollichthys mauli* (Poll, 1953) | 9 | 0.45±0.22 | 6.74 |
| Phosichthyidae | *Vinciguerria attenuata* (Cocco, 1838) | 1 | 0.02±0.02 | 1.12* |
| Phosichthyidae | *Vinciguerria nimbaria* (Jordan & Williams, 1895) | 3 | 0.07±0.04 | 3.37 |
| Stomiidae | *Stomias boa ferox* Reinhardt, 1842 | 3 | 0.1±0.07 | 2.25 |
| Synodontidae | *Synodus foetens* (Linnaeus, 1766) | 13 | 0.75±0.26 | 10.11 |
| Synodontidae | *Synodus synodus* (Linnaeus, 1758) | 1 | 0.1±0.1 | 1.12* |
| Synodontidae | *Trachinocephalus myops* (Forster, 1801) | 2 | 0.08±0.08 | 1.12* |
| Synodontidae | Synodontidae spp. | 1 | 0.08±0.08 | 1.12* |
| Paralepididae | *Lestidium atlanticum* Borodin, 1928 | 4 | 0.11±0.07 | 3.37 |
| Paralepididae | *Lestrolepsis intermedia* (Poey, 1868) | 8 | 0.27±0.12 | 6.74 |
| Paralepididae | *Sudis* sp. | 1 | 0.02±0.02 | 1.12* |
| Paralepididae | *Uncisudis* sp. | 1 | 0.02±0.02 | 1.12* |
| Paralepididae | Paralepididae spp. | 3 | 0.07±0.06 | 2.25 |
| Evermannellidae | *Evermannella melanoderma* Parr, 1928 | 1 | 0.03±0.03 | 1.12* |
| Myctophidae | *Hygophum hygomii* (Lütken, 1892) | 2 | 0.04±0.03 | 2.25 |
| Myctophidae | *Hygophum reinhardtii* (Lütken, 1892) | 1 | 0.03±0.03 | 1.12* |
| Myctophidae | *Hygophum* sp. | 1 | 0.04±0.04 | 1.12* |
| Myctophidae | *Myctophum affine* (Lütken, 1892) | 1 | 0.04±0.04 | 1.12* |
| Myctophidae | *Myctophum nitidulum* Garman, 1899 | 7 | 0.44±0.28 | 5.62 |
| Myctophidae | *Myctophum* spp. | 5 | 0.12±0.05 | 5.62 |
| Myctophidae | *Ceratoscopelus warmingii* (Lütken, 1892) | 2 | 0.07±0.05 | 2.25 |
| Myctophidae | *Ceratoscopelus townsendi* (Eigenmann & Eigenmann, 1889) | 34 | 0.91±0.31 | 13.48 |
| Myctophidae | *Ceratoscopelus* spp. | 2 | 0.15±0.13 | 2.25 |
| Myctophidae | *Diaphus garmani* Gilbert, 1906 | 11 | 0.37±0.21 | 4.49 |
| Myctophidae | *Diaphus brachycephalus* Tåning, 1928 | 156 | 6.34±2.55 | 22.47 |
| Myctophidae | *Diaphus mollis* Tåning, 1928 | 66 | 1.96±0.46 | 25.84 |
| Myctophidae | *Diaphus metopoclampus* (Cocco, 1829) | 5 | 0.47±0.32 | 3.37 |
| Myctophidae | *Diaphus* spp. | 4 | 0.16±0.11 | 3.37 |
| Myctophidae | *Lampadena luminosa* (Garman, 1899) | 11 | 0.19±0.14 | 2.25 |
| Myctophidae | *Lampanyctus lepidolychnus* Becker, 1967 | 3 | 0.08±0.05 | 3.37 |
| Myctophidae | *Lampanyctus* sp. 1 | 1 | 0.02±0.02 | 1.12* |
| Myctophidae | *Lampanyctus* sp. 2 | 1 | 0.02±0.02 | 1.12* |
| Myctophidae | *Lampanyctus* spp. | 1 | 0.05±0.05 | 1.12* |
| Myctophidae | *Lepidophanes guentheri* (Goode & Bean, 1896) | 20 | 0.54±0.23 | 8.99 |
| Myctophidae | *Lepidophanes* spp. | 5 | 0.11±0.11 | 1.12* |
| Myctophidae | *Lobianchia gemellarii* (Cocco, 1838) | 1 | 0.05±0.05 | 1.12* |
| Myctophidae | *Lobianchia* sp. | 2 | 0.1±0.1 | 1.12* |
| Myctophidae | *Notolychnus valdiviae* (Brauer, 1904) | 1 | 0.02±0.02 | 1.12* |
| Myctophidae | *Notoscopelus resplendens* (Richardson, 1845) | 4 | 0.11±0.07 | 3.37 |
| Myctophidae | *Taaningichthys minimus* (Tåning, 1928) | 1 | 0.04±0.04 | 1.12* |
| Myctophidae | Myctophidae spp. | 8 | 0.39±0.21 | 7.87 |
| Bregmacerotidae | *Bregmaceros atlanticus* Goode & Bean, 1886 | 3 | 0.05±0.04 | 2.25 |
| Bregmacerotidae | *Bregmaceros cantori* Milliken & Houde, 1984 | 34 | 1.59±0.66 | 10.11 |
| Bregmacerotidae | *Bregmaceros* spp. | 7 | 0.17±0.13 | 2.25 |
| Phycidae | *Urophycis mystacea* Miranda Ribeiro, 1903 | 11 | 0.59±0.3 | 7.87 |
| Merlucciidae | *Merluccius* sp. | 1 | 0.01±0.01 | 1.12* |
| Macrouridae | Macrouridae spp. | 5 | 0.26±0.18 | 4.49 |
| Ophidiidae | *Lepophidium* sp. | 2 | 0.11±0.09 | 2.25 |
| Ophidiidae | *Ophidion* sp. | 1 | 0.02±0.02 | 1.12* |
| Ophidiidae | Ophidiidae sp. | 1 | 0.05±0.05 | 1.12* |
| Bythitidae | Bythitidae sp. | 3 | 0.43±0.43 | 1.12* |
| Lophiidae | Lophiidae sp. | 1 | 0.08±0.08 | 1.12* |
| Melamphaidae | *Poromitra* sp. | 1 | 0.05±0.05 | 1.12* |
| Fistulariidae | *Fistularia petimba* Lacepède, 1803 | 1 | 0.13±0.13 | 1.12* |
| Scorpaenidae | *Pontinus rathbuni* Goode & Bean, 1896 | 2 | 0.08±0.06 | 2.25 |
| Scorpaenidae | Scorpaenidae sp. 1 | 6 | 0.52±0.29 | 4.49 |
| Scorpaenidae | Scorpaenidae sp. 2 | 1 | 0.03±0.03 | 1.12* |
| Scorpaenidae | Scorpaenidae sp. 3 | 1 | 0.02±0.02 | 1.12* |
| Triglidae | *Prionotus* sp. | 7 | 0.21±0.11 | 5.62 |
| Bramidae | *Brama caribbea* Mead, 1972 | 1 | 0.04±0.04 | 1.12* |
| Serranidae | *Centropristis* sp. 1 | 2 | 0.1±0.1 | 1.12* |
| Serranidae | *Centropristis* sp. 2 | 1 | 0.05±0.05 | 1.12* |
| Serranidae | *Mycteroperca* sp. | 1 | 0.05±0.05 | 1.12* |
| Serranidae | *Rypticus* sp. | 1 | 0.05±0.05 | 1.12* |
| Serranidae | Serranidae sp. | 3 | 0.11±0.11 | 1.12* |
| Carangidae | *Parona signata* (Jenyns, 1841) | 2 | 0.08±0.08 | 1.12* |
| Carangidae | *Trachinotus* sp. | 1 | 0.04±0.04 | 1.12* |
| Carangidae | *Trachurus* sp. | 9 | 0.42±0.4 | 2.25 |
| Carangidae | *Selene* sp. | 1 | 0.04±0.04 | 1.12* |
| Carangidae | Carangidae spp. | 9 | 0.25±0.14 | 4.49 |
| Gerreidae | Gerreidae sp. | 2 | 0.18±0.13 | 2.25 |
| Sciaenidae | *Bairdiella* sp. | 5 | 0.19±0.1 | 4.49 |
| Sciaenidae | *Cynoscion* sp. | 3 | 0.27±0.17 | 3.37 |
| Sciaenidae | *Larimus* sp. | 1 | 0.04±0.04 | 1.12* |
| Sciaenidae | *Micropogonias furnieri* (Desmarest, 1823) | 5 | 0.32±0.18 | 4.49 |
| Sparidae | Sparidae sp. | 1 | 0.07±0.07 | 1.12* |
| Mugilidae | *Mugil* sp. | 2 | 0.09±0.08 | 2.25 |
| Scaridae | *Sparisoma* sp. | 1 | 0.03±0.03 | 1.12* |
| Blenniidae | *Parablennius* sp. | 2 | 0.2±0.14 | 2.25 |
| Blenniidae | *Hypleurochilus fissicornis* (Quoy & Gaimard, 1824) | 2 | 0.14±0.11 | 2.25 |
| Blenniidae | Blenniidae sp. 1 | 2 | 0.11±0.09 | 2.25 |
| Blenniidae | Blenniidae spp. | 1 | 0.15±0.15 | 1.12* |
| Gobiidae | *Bathygobius soporator* (Cuvier & Valenciennes, 1837) | 1 | 0.02±0.02 | 1.12* |
| Gobiidae | *Ctenogobius boleosoma* (Jordan & Gilbert, 1882) | 17 | 1.63±0.84 | 6.74 |
| Gobiidae | Gobiidae spp. | 11 | 0.97±0.37 | 10.11 |
| Sphyraenidae | *Sphyraena barracuda* (Edwards, 1771) | 6 | 0.3±0.19 | 5.62 |
| Sphyraenidae | *Sphyraena tome* Fowler, 1903 | 1 | 0.1±0.1 | 1.12* |
| Sphyraenidae | *Sphyraena* spp. | 7 | 0.2±0.1 | 4.49 |
| Gempylidae | *Nealotus tripes* Johnson, 1865 | 1 | 0.01±0.01 | 1.12* |
| Trichiuridae | *Trichiurus lepturus* Linnaeus, 1758 | 17 | 0.81±0.29 | 10.11 |
| Scombridae | *Auxis* sp. | 11 | 0.3±0.14 | 5.62 |
| Scombridae | *Euthynnus alletteratus* (Rafinesque, 1810) | 45 | 1.01±0.62 | 7.87 |
| Scombridae | *Katsuwonus pelamis* (Linnaeus, 1758) | 1 | 0.03±0.03 | 1.12* |
| Scombridae | *Scomber colias* Gmelin, 1789 | 5 | 0.27±0.16 | 5.62 |
| Scombridae | *Thunnus* spp. | 14 | 0.69±0.39 | 5.62 |
| Scombridae | Scombridae spp. | 3 | 0.07±0.05 | 2.25 |
| Bothidae | *Bothus* sp. | 1 | 0.05±0.05 | 1.12* |
| Bothidae | *Monolene antillarum* Norman, 1933 | 1 | 0.05±0.05 | 1.12* |
| Bothidae | Bothidae sp. | 1 | 0.08±0.08 | 1.12* |
| Paralichthyidae | *Citharichthys* sp. | 3 | 0.25±0.18 | 3.37 |
| Paralichthyidae | *Etropus longimanus* Norman, 1933 | 1 | 0.1±0.1 | 1.12* |
| Cynoglossidae | *Symphurus plagiusa* (Linnaeus, 1766) | 6 | 0.25±0.22 | 2.25 |
| Cynoglossidae | *Symphurus trewavasae* Chabanaud, 1948 | 1 | 0.03±0.03 | 1.12* |
| Cynoglossidae | *Symphurus kyaropterygium* Menezes & Benvegnú, 1976 | 4 | 0.27±0.23 | 2.25 |
| Cynoglossidae | *Symphurus* spp. | 6 | 0.28±0.19 | 3.37 |
| Molidae | *Mola mola* (Linnaeus, 1758) | 1 | 0.02±0.02 | 1.12* |
| Yolk sac larvae | Unidentified | 175 | 12.07±3.31 | 40.45 |
| Yolk sac larvae | Leptocephalus | 2 | 0.07±0.05 | 2.25 |
| Unidentified | Unidentified larvae | 176 | 8.66±1.87 | 61.80 |
| Anguilliformes | Unidentified eggs | 13 | 0.44±0.2 | 6.74 |
| Engraulidae | Unidentified eggs | 408 | 39.54±22.14 | 12.36 |
| Sternoptychidae | *Maurolicus muelleri* (Gmelin, 1789) | 57 | 4.87±4.21 | 6.74 |
| Unidentified | Unidentified eggs | 1452 | 116.71±39.76 | 71.91 |

*Species/taxa caught in only one sample.
